# Health Supervision in Schools

**Published:** 1913-08

**Authors:** L. W. Sackett

**Affiliations:** Instructor in the Philosophy of Education, the University of Texas


### Health Supervision in Schools.

BY L. W. SACKETT, PH. D., INSTRUCTOR IN THE PHILOSOPHY OF EDUCATION, THE UNIVERSITY OF TEXAS.

Why should it be necessary to discuss the problem of health inspection in schools? Surely the question is beyond the experimental stage in the communities where it has been adopted. Yet many people are actively opposed to it, and a still greater number are unwilling to advocate it openly. The pernicious doctrine of the divine right of the parent to have exclusive care of the health of the child stands in the way of the welfare of both the parent and the child. It is not uncommon to hear the complaint that the schools are in this respect exercising, or are attempting to exercise, undue paternalism—a type of argument consistent with the age when disease was thought to be providentially sent. Then, also, the term "medical inspection" has given some people a vision of a dignified doctor going down the row of seats under the autocratic authority of the teacher, forcing every child to swallow his portion of a patent nostrum from a communal spoon. We should all object to that or anything like it. Let us use the term *"health supervision"* instead of medical inspection. It gives a truer perspective to the work. The medical profession may be called upon as inspectors and supervisors, but the chief function of these representatives will be to ferret out difficulties and to institute preventive measures; not primarily to administer treatment even to those already affected. Parents may attend to the treatment through the family physician, if they so desire. The duty of the inspector will be to guard the health of all the children in the school. This is true health supervision. You mothers who object to this as paternalism, consider how you would view it if a neighbor's child were about to infect your own with some loathsome or deadly disease. I would even appeal to your selfish interest to secure what the schools so seriously need.

We are going to have health supervision in all the schools sooner or later. Such is the import of the new health consciousness now emerging. It is only a matter of what State or community will be the last to fall into line. To have compulsory education without proper sanitary inspection is a crime against childhood. Of course, we shall all eventually have compulsory education and then look back and smile at our old narrow conservatism. But even when the child goes to school without State laws to compel him to do so the health problem is the same. We are face to face with a new order of affairs in child development. Civilization and book education have come as a biological catastrophe to the child. Entirely new problems are rising every day. The young child caught wild and confined in a small seat of a small school room during the most vitalizing part of the day has his sensitivity to disease greatly heightened. Keepers of zoological gardens report very high mortality when animals are captured from a wild state. Many will not survive the early confinement. Many of those taken young grow to be stunted specimens, even with competent veterinary care. The child may not really die as a result of school conditions, but it is often appropriate to quote that sad line from Kipling: "A part of him lives but the most of him dies." This should not be,—it must not be. The schools must not only not injure the health of the child, but they must strive as assiduously to promote his physical vigor as they are now striving to promote his mental development. The teacher is obviously incapable of performing both these functions well, and consequently the need for health supervision is apparent. Even when the teacher feels that something is wrong; the attempt on her part to remedy it would result only in unpleasantness. The health supervisor has the authority to cause the trouble to be set right.

But I do not wish to seem to be arguing the question. Properly presented, it is too obvious to need argument. Let us see what the conditions are as actually revealed in the places where health supervision is carried on most perfectly. European countries have done more than other countries in this line of philanthropic efforts, Switzerland leading all others. The work was begun in this country less than twenty years ago. The State of Massachusetts has the most efficient system of any State in the Union. At present in that State, where a large percentage of the population is urban, 98 per cent of the children in cities and towns have the benefit of health supervision. We are assured in a report from there that "succeeding generations will be less burdened by physical and mental cripples, will be free from contagious and infectious diseases, and will have a more intelligent conception of disease, and its cause and effect." It is also gratifying to learn that this is accomplished at the nominal cost of 20 cents per child for the whole State, though in Boston where the work is most efficient the cost is considerably greater. Out of a school attendance of approximately 400,000 in Chicago, in 1909, a corps of 100 medical health officers and 41 nurses made over 240,000 examinations, finding 123,000 or 51 per cent of those examined to be defectives. Fort Worth, Texas, in 1910, referred for treatment 710 out of a thousand examinations. Houston, in 1910-11, excluded 38 children with trachoma and 74 with developing symptoms of the same disease. In the same city there has recently been established a free eye, ear, nose, throat and dental clinic for school children. Cleveland, Ohio, discovered in the school room and excluded during the years 1910-12 no less than 48 cases of tuberculosis, 155 of tonsilitis, 6 of trachoma, 6 of diphtheria, 35 of scarlet fever, 17 of measles, 34 of chickenpox, and 41 of mumps. Such an array of contagious diseases in the school room is appalling. It is not only "food for thought" but is surely "food for very prompt and decisive action." Coupled with the fact that many cases were found where the child himself was not affected by the germs of the disease but was carrying them and spreading them unconsciously to his playmates, the problem of intercepting contagious diseases becomes even more serious. How can a child have health in such an environment? The constant strain of throwing off infection is weakening to both body and mind, and may account for many of Edward Bok's fifty thousand children which he estimates are made nervous wrecks every year in the schools. According to general custom, the family physician is not called until the case is well advanced. Then it is generally too late to prevent the spread of infection, and most certainly too late to save the child from the ravages of the disease. The health supervisor should find the case in its incipient stage and save both the child and the community.

Not only are contagious diseases a serious problem, but physical defects are a problem of no less importance. Most physical defects yield to treatment if taken in time, but they may be a serious handicap while they are present. To illustrate with a single instance, it has been found that more than 13 per cent of the "repeaters" in the schools belong to this class. Among the defective conditions commonly found, diseased tonsils, adenoids, swollen glands and nasal obstructions hold a prominent place. Nearly 50,000 cases of these defects were found in Cleveland alone in the two years 1910-12. Defective vision was discovered in nearly 18,000 and defective hearing in 2500 cases. The number of cases fully corrected in this city was 5373, while many more were greatly improved. "Of the corrected cases, approximately two-thirds of the number were repeaters, presumably by the reason of correctable defects." The extra cost of taking care of the unnecessary laggards and repeaters, together with the cost of suspending school in case of an epidemic, would give health supervision to all the children.

Statistics show that 70 to 90 per cent of all children in the public schools have defective teeth. In the home, this is too often considered a small matter. Both the child and the parents need to be educated to the fact that these pockets of decayed teeth are natural breeding places for bacteria of diphtheria, pneumonia and tuberculosis. They are merely small garbage cavities which are seldom cleaned and never disinfected. Some children who have good teeth come to school with mouths as filthy as garbage cans, and some parents exercise small precaution and set no better personal examples at home. Human teeth would seem to be deteriorating under the chemical action of highly seasoned food. However that may be, the question is one of great economic importance, as careful supervision of dental conditions, followed by the necessary repair work up to the end of the twelfth year, would prevent most of the difficulty of later years. This, of course, can be done without school supervision, but there is not sufficient assurance that it will be. Serious difficulties should be anticipated and avoided. When a mother is told by the visiting nurse who follows the case into the home that her child is being retarded by bad teeth, she may not be so prone to lay the blame on the personal spite of the teacher.

The operation of this same principle in a wider sense is seen by the effect of life insurance examinations on vital statistics. Many a man's life has been saved because some life insurance company refused to carry him as a risk. His true physical condition was revealed to him by experts before he was conscious of it himself, and in time for treatment to be effective. Delay would have meant death. Even an adult is often the poorest judge of his own physical condition. How much less would children be able to know about their physical fitness? The family physician could, of course, diagnose the case if it were properly brought before him, but, as indicated above, very often he has no such opportunity. The expert in health supervision supplements this personal consciousness, and is to all the children what the life insurance examination often is to the adult.

But the greatest of all benefits to be derived from health supervision is the development of hygienic personal life habits. Most of our adult daily life is spent in carrying out the habits formed in childhood. Many of the world's authorities on hygiene regularly violate every tenet of their teaching because their behavior is determined by habits formed before they become specialists. Knowledge of conditions is not a sufficient guarantee of proper conduct. The teaching and the practice of hygiene should be emphasized in the plastic age of the child and his school education should never be completed without a physical expression of his knowledge of personal hygiene in the habits of his daily life. Thus health supervision becomes one of the most personal phases of the child's education, and should, through the co-operation of teachers and parents, become an effective agency in developing permanent health habits and health control in the citizens-to-be who are now in our schools. If this be paternalism, and if paternalism is necessary to accomplish this result, then pray let us have more paternalism!

Health supervision is a new and technical art. The schools should be the first and not the last to recognize and foster it. It is not safe to leave so important a matter to the caprices of the individual. Let the mothers of the land rise up and demand that the health of the child and his training in hygienic habits of living be as carefully supervised as are his instructions in curricular subjects and his moral training. Indeed, in many cases, it would be found that the difficulties both in intellectual progress and moral constraint are solvable only in the light of facts revealed by the health supervisor. The so-called "dullard" and the "trouble-maker" may be in far greater need of a competent physician and surgeon than of a tutor and a disciplinarian. The case in the general field is well put in a recent Bureau of Education bulletin (Bull., 1913, No. 18, p. 7), which might be taken as the creed of the health inspector of schools: "When people have pure food, pure water, pure air, and are freed from the dust of houses, streets and manufacturing industries; when they have good light and abundant sunshine, sanitary houses, barns and outbuildings; when they are protected from germ-carrying agencies, such as flies, mosquitoes, rats, mice, and all such pests; when they are protected from people who are carriers of disease germs, and taught how to disinfect their homes and communities; when they are taught to work and play, eat and sleep, dress and bathe, according to the laws of health; when they learn to care for their teeth and their eyes, the main problem of hygienic living will be solved and human life relieved of its greatest sources of suffering and disease."

